# Bouganin, an Attractive Weapon for Immunotoxins

**DOI:** 10.3390/toxins10080323

**Published:** 2018-08-08

**Authors:** Massimo Bortolotti, Andrea Bolognesi, Letizia Polito

**Affiliations:** Department of Experimental, Diagnostic and Specialty Medicine-DIMES, General Pathology Section, Alma Mater Studiorum—University of Bologna, Via S. Giacomo 14, 40126 Bologna, Italy; massimo.bortolotti2@unibo.it (M.B.); letizia.polito@unibo.it (L.P.)

**Keywords:** antiviral activity, Bougainvillea, bouganin, cancer therapy, immunotherapy, immunotoxins, ribosome-inactivating proteins, rRNA *N*-glycosylase activity, VB6-845

## Abstract

Bougainvillea (*Bougainvillea spectabilis* Willd.) is a plant widely used in folk medicine and many extracts from different tissues of this plant have been employed against several pathologies. The observation that leaf extracts of Bougainvillea possess antiviral properties led to the purification and characterization of a protein, named bouganin, which exhibits typical characteristics of type 1 ribosome-inactivating proteins (RIPs). Beyond that, bouganin has some peculiarities, such as a higher activity on DNA with respect to ribosomal RNA, low systemic toxicity, and immunological properties quite different than other RIPs. The sequencing of bouganin and the knowledge of its three-dimensional structure allowed to obtain a not immunogenic mutant of bouganin. These features make bouganin a very attractive tool as a component of immunotoxins (ITs), chimeric proteins obtained by linking a toxin to a carrier molecule. Bouganin-containing ITs showed very promising results in the experimental treatment of both hematological and solid tumors, and one bouganin-containing IT has entered Phase I clinical trial. In this review, we summarize the milestones of the research on bouganin such as bouganin chemico-physical characteristics, the structural properties and de-immunization studies. In addition, the in vitro and in vivo results obtained with bouganin-containing ITs are summarized.

## 1. Introduction

Ribosome-inactivating proteins (RIPs) are a family of plant proteins characterized by an enzymatic activity classically identified as rRNA *N*-glycosylase (EC 3.2.2.22). These enzymes, which are widely distributed among plant genera, specifically remove the A4324 adenine residue of the 28S rRNA in rat ribosome thus interfering with the ribosome/elongation factor 2 interaction, damaging ribosomes in an irreversible manner and causing the inhibition of protein synthesis [1]. RIPs also show *N*-glycosylase activity on different other substrates, such as mRNA, tRNA, DNA and poly(A) [2,3]. As regards the structure, RIPs can mainly be divided into two groups: type 1, consisting of a single-chain protein with enzymatic activity, and type 2, consisting of an enzymatic A-chain linked to a B-chain with lectin properties. The presence of the B-chain allows the fast internalization of the toxin into the cell, so conferring to most type 2 RIPs a very high toxicity [4]. RIPs can kill the cells by apoptosis [5,6], even if other cell death mechanisms are involved in the pathogenesis of RIP intoxication [7,8]. It is worth noting that RIPs are also able to depurinate viral nucleic acids. In fact, many RIPs can inhibit animal and plant viruses through mechanisms that have not yet been fully clarified. There is evidence that the antiviral action of RIPs cannot be solely attributed to the inhibition of ribosomes, but also to direct interaction of RIPs with viral RNA or DNA [2,9].

Since 1925, it was known that leaf extracts of some plants were able to prevent the infection of other plant species when mixed with a suspension of tobacco mosaic virus (TMV) [10]. Those observations allowed the partial isolation of the antiviral principle of *Phytolacca americana* [11] and *Dianthus caryophyllus* [12]. Afterwards, the antiviral activity was attributed to specific proteins (named pokeweed antiviral protein (PAP) and dianthin, respectively). Moreover, it was shown that also the protein synthesis inhibition activity, present in the extracts, was due to the same proteins [13,14,15]. It was subsequently established that most of the tested RIPs, including type 2 ones, were able to prevent infection with TMV in *Nicotiana benthamiana* leaves, albeit at different concentrations. Plant extracts with antiviral activity did not prevent the infection of autologous plants but were effective only on heterologous plants. This led to the conclusion that the antiviral principles acted on the plant, rather than on the viruses. Further studies showed that the in vitro antiviral activity of RIPs could also be directed against animal viruses, both RNA and DNA viruses [9].

In medicine, RIPs found application as toxic moiety of conjugates, chimeric molecules specifically targetable to unwanted cells responsible for pathologic conditions. Conjugates containing RIPs linked to monoclonal antibodies (mAbs) or their fragments are referred to as immunotoxins (ITs). ITs can be obtained both by the chemical linkage of the toxic moiety to mAbs and by genetic engineering to obtain recombinant conjugates [16]. RIP-containing ITs have been included in many clinical trials against various diseases, often achieving promising results, especially in the treatment of hematological neoplasms [17].

## 2. Purification and Antiviral Properties of Bouganin

*Bougainvillea spectabilis* Willd., also known as “paper flower” or “Bougainvillea”, is a woody vine belonging to Nyctaginaceae family. It is native to South America but spread all over the world for its ornamental characteristics. This plant, in fact, is frequently blooming and its bracts have an intense purple or magenta color. 

As for many other RIP-containing plants [18], Bougainvillea has long been used as medicinal plant, mainly in Latin America and Mexico [19]. The extracts from several plant tissues, mainly leaves, flowers and stem barks, are utilized in traditional medicine in forms of infusions, decoctions and tinctures. Drunk as a tea, Bougainvillea extracts are employed against cough, sore throat, flu, fever, diarrhea, diabetes, hepatitis and liver problems, asthma, bronchitis, to reduce stomach acidity, dissolve blood clots, regulate menstruation and stop leucorrhea, and for anemia associated with gastrointestinal bleeding and epigastric pain. Infusion of flowers is drunk as a remedy for low blood pressure [20].

Several studies have been conducted in order to evaluate the pharmacological activities of phytochemical constituents isolated from different Bougainvillea tissues. Experimental evidences showed that such molecules can exert antibacterial, antihyperlipidemic, antidiabetic, antifertility, antioxidant, anti-inflammatory, and antiulcer activities [21].

The first experimental evidences of antiviral effect of Bougainvillea date back to the 80s when it was evidenced that the infection of tobacco plants by TMV was prevented by leaf extracts [22,23]. The prevention of the infection was attributed to protein factors.

**Figure 1:**
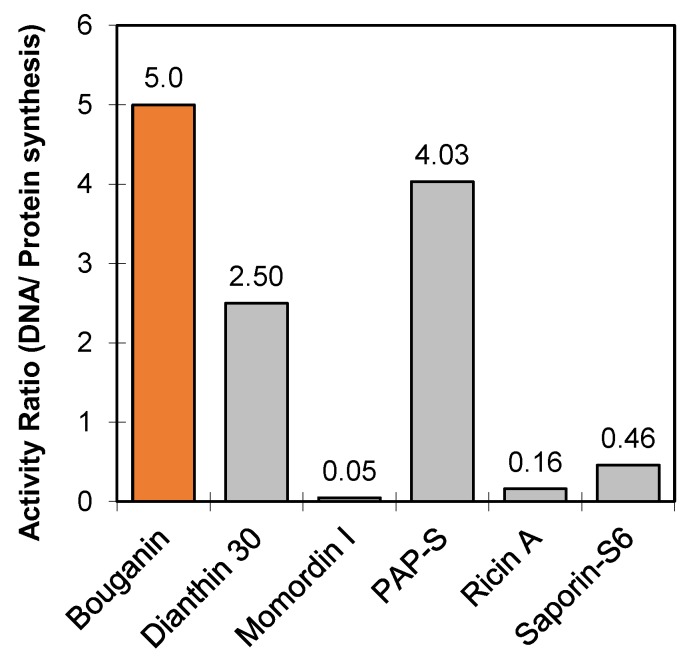
Activity ratio of bouganin compared to other type 1 RIPs and ricin A chain. The bar values represent the ratio between the activity on hsDNA and the activity on cell-free protein synthesis, expressed as 10^3^ U/mg, as reported in Table 1.

In 1997, Bolognesi and co-workers identified for the first time the presence of type 1 RIPs in the leaves of Bougainvillea. At least seven different RIPs were purified by ion-exchange chromatography of leaf extracts. The authors’ attention focused on the first eluted pick, corresponding to a protein that was named bouganin. This protein was chosen for further experiments because it had the highest specific inhibitory activity on cell-free protein synthesis and gave the highest yield after purification. Bouganin has the properties of type 1 RIP, in that it: (i) is a single-chain protein with a molecular mass of about 30 kDa and an isoelectric point in the alkaline region; (ii) inhibits protein synthesis in a cell-free system (IC_50_ 10 ng/mL) despite its lower activity on whole cells, compared to other type 1 RIPs; (iii) has *N*-glycosylase activity; and (iv) has antiviral activity. Moreover, it appeared to be homogeneous at 99% by reverse-phase High Performance Liquid Chromatography (HPLC) analysis, and on Sodium Dodecyl Sulphate-PolyAcrylamide Gel Electrophoresis (SDS-PAGE) gave a single band with mobility corresponding to Mr 26200. The RIP released a single adenine residue from rat liver ribosomes but several tens of adenine residues from other substrates as rRNA from E. coli, viral RNA, poly (A) and several hundreds of adenine residues from herring sperm DNA (hsDNA), thus possessing a polynucleotide:adenosine glycosidase activity [24]. As reported in Table 1 and Figure 1, bouganin showed the highest activity ratio compared to other type 1 RIPs and to ricin A chain. This represents an advantage because the RIP can exert its activity on different substrates, triggering cell death through multiple pathways.

Bouganin prevented systemic infection by artichoke mottled crinkle virus of *Nicotiana benthamiana* plants, presumably by inhibiting viral replication at the site of infection. Despite the high specific enzymatic activity, bouganin showed a very low toxicity for animals (mice); no mouse was killed by a dosage of 32 mg/kg of bouganin; this dose was very toxic for many other type 1 RIPs. Certainly, bouganin appears to be one of the least toxic RIPs, in comparison with other well-known RIPs (Table 1) [25].

## 3. Recombinant Bouganin and Structure/Function Studies

In 2002, den Hartog and co-workers cloned, expressed, purified, and characterized the recombinant bouganin. The cDNA, synthetized from total RNA isolated from Bougainvillea leaves, encoded for a precursor protein of 305 amino-acid residues. On the basis of the bouganin primary sequence, it was found that 26 residues at the amino-terminal and 29 residues at the carboxy-terminal are post-translationally removed to produce the mature form, which consists of 250 amino acids. The 26-residue leader portion is a secretory signal sequence rich in hydrophobic amino acids, which probably direct the transport of the nascent polypeptide chain across the endoplasmic reticulum membrane into the endoplasmic reticulum lumen. Bouganin and the other type 1 RIPs have little or no homology in the *C*-terminal cleaved amino-acid sequence. This demonstrates that these sequence motives, of unknown function, are not conserved in nature [30]. The recombinant molecule had similar enzymatic activity in a cell-free protein synthesis assay and had comparable toxicity on cells as compared to native bouganin [30].

**Figure 2:**
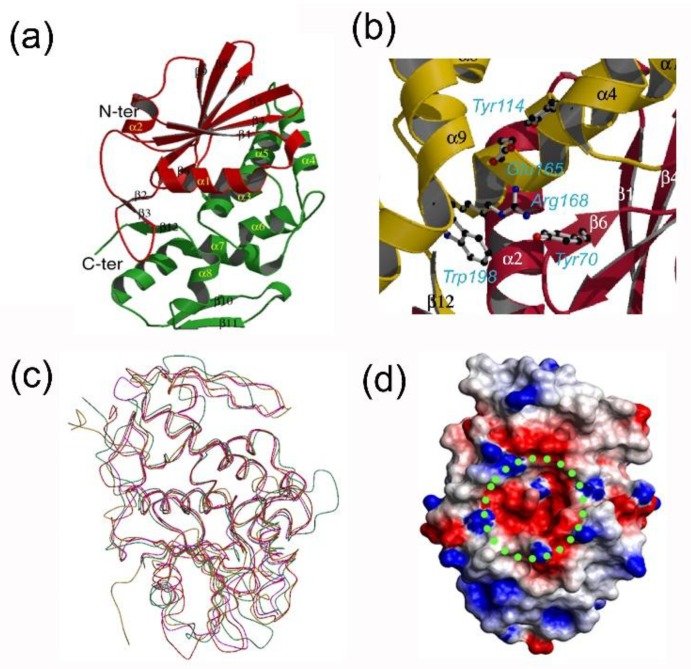
(**a**) Ribbon model of the crystal structure of bouganin (accession number Protein Data Bank 3CTK). The *N*-terminal and the *C*-terminal domains are colored in red and green respectively; (**b**) Catalytic site of bouganin. The conserved important residues are showed in ball-and-sticks; (**c**) Structural comparison between different type 1 RIPs and ricin A chain. Superimposition of the Cα atoms of bouganin (magenta), dianthin 30 (green), PAP-R (red) and ricin A chain (gold). The figures were produced by MOLSCRIPT [33] and rendered by RASTER3D [34]; (**d**) Electrostatic surface potential of bouganin surface at pH 7. The positive regions are represented in blue and the negative ones are colored in red. The active pocket is highlighted by a green circle. The figure was produced by GRASP [35].

Subsequently, the three-dimensional structure of bouganin was solved, demonstrating that its overall structure is like other RIPs, maintaining the typical RIP fold [26]. The *N*-terminal domain (in red in Figure 2a) is made up of a mixed β-sheet of seven strands (β1–β9). The five central strands run antiparallel and the other extern four are parallel to the neighbors. The α2 and the α3 helixes are connected to short structural motifs of β strands that are respectively the first two and the last two of the sheets. In bouganin, the *C*-terminal domain (in green in Figure 2a) is mainly composed of eight α-helices. This region shows a loop, flanking helix α9, composed of two antiparallel β-strands connected by a short helix. By site-directed mutagenesis experiments in RIPs, it has been possible to identify five highly conserved residues, which are involved in *N*-glycosylase activity and correspond to Tyr70, Tyr114, Glu165, Arg168 and Trp198 in bouganin [31]. (Figure 2b). Moreover, Phe169, another highly conserved amino acid near the active site, could play a role in stabilizing the conformation of Arg168 side chain [32].

Similarly to other RIPs, the active site is in a cleft at the center of the molecule. The authors suggested an explanation for the lower bouganin activity based on one amino acid substitution. In fact, the Asn78 in ricin or the corresponding Asn70 in PAP-R that is proposed to be involved in the interactions with the substrate, probably with the phosphodiester group between the target adenosine and the subsequent guanosine, was conserved in most considered RIPs except in bouganin, which has an aspartate in the corresponding position [26]. The backbone super-imposition of bouganin demonstrates that the central area of the molecule is well super-imposable, while big differences are reported in the peripherical portions (Figure 2c). Analyzing bouganin electrostatic surface potential, it is evidenced that the active site is a wide negative cavity, except for a small positive zone determined by the catalytic residue Arg168 (Figure 2d).

## 4. Antigenic Properties of Bouganin

Despite the encouraging results obtained with RIP-containing ITs in the treatment of hematological tumors (see Introduction), about 40% of patients respond producing anti-RIP antibodies [36]. This obstacle can be bypassed through multiple treatments with ITs containing different RIPs that do not cross-react each other [37]. Nevertheless, it is crucial to identify the most critical epitopes on RIPs to reduce their immunogenicity and thus improve their therapeutic utility. In this sense, Cizeau and co-workers undertook pioneering work synthetizing 89 peptides of 15 amino acids, each with an overlap of 12 residues, covering the mature bouganin protein. Selecting the peptides that induce T-cell proliferation, through in silico analysis, the residues presented as antigenic determinants for major histocompatibility complex (MHC) class II recognition were identified. The authors expressed a bouganin mutant, called de-bouganin, carrying four mutations (Val123Ala, Asp127Ala, Tyr133Asn, Ile152Ala) that did not affect the catalytic activity of the enzyme but significantly reduced the immune response of the host versus the toxin [38].

Analysis of the primary structure of wild-type bouganin compared to those of some type 1 RIPs (namely, saporin-SO6, tricosanthin and alpha-momorcharin) shows that a Tyr-Tyr-Phe (YYF) or Tyr-Phe-Phe (YFF) sequence is present in all the toxins except bouganin. It has been claimed that YYF/YFF sequences are recognized by different MHC class II alleles, thus justifying the higher antigenicity of saporin-S6, tricosanthin and alpha-momorcharin than bouganin [39]. The Lys-Arg (KR) motif, already identified in trichosanthin as responsible of its immunogenicity, is not significantly represented in conserved positions. Moreover, the bouganin residues inducing immunological responses (Val, Asp, Tyr and Ile) are not conserved amongst this group of type I RIPs [39]. These characteristics make bouganin a RIP with completely different antigenic properties from other type 1 RIPs. In effect, if tested with sera against six other type 1 RIPs, namely saporin-S6, and dianthin 32 (Caryophyllaceae), PAP-R (Phytolaccaceae), momordin I, momorcochin-S and trichokirin (Cucurbitaceae), bouganin gave no cross-reaction with any tested antiserum [24].

## 5. Bouganin-Containing Immunotoxins, Preclinical Evaluations

Bouganin represents a good candidate as toxic moiety to obtain ITs because of its high activity ratio, very low aspecific toxicity reported in animal models and high stability to derivatization and conjugation procedures [24].

The first bouganin-containing ITs were constructed conjugating bouganin to the M24 mAb (anti-CD80) and to the 1G10 mAb (anti-CD86) and comparing them to ITs built with the same mAbs and two different type 1 RIPs, gelonin, and saporin [27]. Bouganin incremented its toxicity on target cells by 3–4 log upon conjugation, as measured by inhibition of protein synthesis with IC_50_s ranging from 4.61 to 192 pM as RIPs, comparable with IC_50_ values obtained with gelonin-containing ITs and 1–2 logs higher than saporin-containing ITs built both with the same mAbs and with another anti-CD80 IT [27,40]. All the anti-CD80 ITs resulted in higher cytotoxic effect than anti-CD86 ones, probably reflecting a higher antigen affinity and/or better internalization. This difference was more evident for bouganin-containing ITs. Moreover, bouganin-containing ITs induced apoptosis in target cells and they did not significantly affect the recovery of committed progenitors at concentrations up to 100 nM [27].

Recombinant ITs have the advantage to be stably formulable and less immunogenic than chemically linked ITs, so they are generally more suitable for clinical use. In 2009, Cizeau and co-workers genetically linked de-bouganin to an anti EpCAM Fab moiety via a peptidic linker to create the fusion construct VB6-845. This conjugate bound and selectively killed EpCAM-positive cell lines with a greater potency than many commonly used chemotherapeutic agents. In vivo efficacy was demonstrated using an EpCAM-positive human tumor xenograft model in severe combined immunodeficiency (SCID) mice; the majority of treated mice being tumor free at the end of the study [38]. The same research group described the intracellular trafficking in EpCAM-positive cells of VB6-845. De-bouganin recombinant IT was shown to co-localize along with the EEA1 endosomal and LAMP-2 lysosomal markers after 15 and 45 min whereas it did not traffic via a Golgi/ER pathway in contrast to ricin [41]. The preclinical evaluation of safety and suitability of VB6-845 as a systemically administered drug for the treatment of solid tumors was performed in animal models [42,43]. Efficacy studies in mice bearing human tumors demonstrated that VB6-845 specifically and potently targeted EpCAM-positive tumors. SCID mice bearing subcutaneous ovarian tumor (NIH:OVCAR-3 cells) were treated with 10 and 20 mg/kg VB6-845 IT. All mice were alive at the end of the study and no animal reached the endpoint tumor volume (750 mm^3^). In HRLN nu/nu mice bearing subcutaneous MCF-7 VB6-845 at 20 mg/kg gave a 70% survival rate over the study period, with 3 mice achieving a complete tumor regression with no measurable tumor mass. The aspecific toxicity was evaluated in animal models, in dose-ranging studies. In rats, single doses until 100 mg/kg of the IT were well tolerated resulting in no-observable adverse effects, but doses of 200 mg/kg caused mild clinical signs that included excessive licking of forepaws, reddened skin on fore and hind paws, edema of the forepaws, and a slight decrease in activity level. In Cynomolgus monkeys, two treatments (+1 and +8 day) for total doses of the IT until 180 mg/kg were well-tolerated when given as a 3-h infusion mimicking the intended route of administration in the clinic; only mild and transitory clinical side effects were reported. In addition, VB6-845 proved to be minimally immunogenic in monkeys. No immune response was observed against either the humanized Fab or the toxin moiety on day 7; however, antibody responses were detected by day 14, being the reactivity to de-bouganin 3-fold lower than to the Fab fragment.

Several type 1 RIP-containing ITs were designed to target growth factor receptors on human tumors, indicating the validity of the idea [44,45,46,47]. In 2016, Dillon and co-workers conjugated de-bouganin to the anti-HER2 mAb trastuzumab. This IT demonstrated greater in vitro potency on HER2-positive cell lines and higher toxicity against tumor cells with cancer stem cell properties than the conjugate containing the tubulin inhibitor mertansine conjugated to trastuzumab. In addition, the authors demonstrated that, unlike for mertansine-trastuzumab IT, T-DM1, the cytotoxic effect of de-bouganin-trastuzumab was not influenced by MK571, an inhibitor of efflux pumps, which is responsible for multidrug resistance [48]. Similarly, ABT-737, a Bcl-2 family inhibitor, modulated T-DM1, but not de-bouganin-trastuzumab cytotoxicity [48]. In the same year, Chooniedass and co-workers described the engineering and biological activity of de-bouganin genetically linked to an anti-HER2 C6.5 diabody (deB-C6.5-diab). On breast cancer cell lines, the DeB-C6.5-diab and de-bouganin-trastuzumab conjugates showed greater cytotoxic activity than auristatin E- and emtansine-trastuzumab conjugates, demonstrating that de-bouganin is effective against tumor cell resistance mechanisms selected in response to immunoconjugates composed by anti-microtubule agents [49]. Main results obtained in preclinical experiments are summarized in Table 2.

## 6. Bouganin-Containing Immunotoxins, Clinical Evaluations

In 2007, VB6-845 entered a Phase I clinical trial (NCT00481936) sponsored by VIVENTIA Biotech Inc. (Winnipeg, MB, Canada). The purpose was to determine the maximum tolerated dose of VB6-845 and to evaluate its safety and tolerability when administered as intravenous infusion once weekly for 4 weeks to patients with advanced solid tumor of epithelial origin [43]. Fifteen neoplastic patients with cancers affecting kidney, ovary, breast, stomach, pancreas, lung, and colon, were enrolled into the study. The tested dosages were 1.0, 2.0 and 3.34 mg/kg. The maximum treatment duration was 16 weeks. Only one case of dose-limiting toxicity was reported. It was a grade 4 acute infusion reaction, which occurred in a patient with metastatic renal cell carcinoma treated with IT at 2.0 mg/kg that showed hypotension and weakness during the third infusion. These reactions were resolved without consequences after just one day of therapy. Five subjects reported serious adverse events: two of them were reported as related to study treatment, they were infusion reactions consisting of a symptom complex characterized by hypotension, fever, and nausea, weakness, drowsiness, chills, and face and neck hyperemia. The study terminated for corporate reasons unrelated to safety and efficacy of the IT. The adverse events reported in the literature are those available in the clinical database at the moment of the early closure of the trial. Exploratory efficacy data revealed encouraging preliminary results. Seven subjects who completed one full cycle (4 weeks) of treatment showed stable disease using standard imaging techniques. For five of them was reported a stable disease 1 week after the completion of the fourth dose. Amongst the three subjects who continued to receive study treatment after the first cycle, one subject had stable disease at the completion of second (8 weeks) and third (12 weeks) cycles. In addition, one patient with renal cell carcinoma and one patient with breast carcinoma had a reduction of the tumor mass [43].

The validity of the bouganin de-immunization approach was assessed on plasma samples from patients treated with VB6-845 IT, testing their immune responsiveness against both humanized Fab and de-bouganin. After 2 weeks from the treatment, no patient plasma samples showed a detectable immune response. After 3 weeks, only 1 patient showed a moderate anti de-bouganin titer, whereas six of seven patients showed anti-Fab titers. By week 4, anti-Fab titers were detectable in all patients, whilst only two patients had barely measurable anti-de-bouganin titers [43].

## 7. Conclusions

The plant toxin bouganin can represent an attractive weapon to construct ITs for the experimental therapy of human neoplasia. In fact, it shows interesting features with respect to other RIPs, such as a high ratio of activity on DNA to that on ribosomal RNA and low systemic toxicity. Moreover, the absence of cross-reactivity with sera against RIPs from other taxonomically related or unrelated plants can represent a useful property for circumventing the immune response after repeated administration of ITs. The sequencing of bouganin and the knowledge of its three-dimensional structure allowed to obtain a not immunogenic mutant of bouganin for clinical use. An IT containing modified bouganin showed encouraging results in Phase I clinical trials on patients with advanced carcinoma. The lack of immune responsiveness towards bouganin in patients illustrates the validity of the T cell epitope-depletion approach to dampen the immune response and strongly supports the utility of bouganin as a cytotoxic payload for systemic delivery.

## Figures and Tables

**Table 1:** Adenine: Polynucleotide glycosidase activity, protein synthesis inhibitory activity and toxicity of bouganin compared with different type 1 RIPs and ricin A chain.

RIP	Adenine Released		Protein Synthesis		Toxicity
	hsDNA ^1^ pmol	Rat Ribosomes ^1^ pmol	Cell Free ^1^ 10^3^ U/mg *	Raji Cells ^2^ IC_50_ (nM)	Mouse ^3^ LD_50_ (mg/kg)
Bouganin	377.7	4.8	75	839	>32 ^4,#^
Dianthin 30	239.9	5.7	96	541	14
Momordin I	27.1	3.9	526	n.a. ^§^	7.4
PAP-S	503.2	5.1	125	n.a. ^§^	2.6
Ricin A chain	48.5	6.2	300	200 ^5^	16 ^6^
Saporin-S6	376.1	19.1	813	23.6	4

^1^ Data from [26]. * One unit of inhibitory activity (U) is defined as the amount of protein causing 50% inhibition in 1 mL of reaction mixture. ^2^ Data from [27]. ^#^ No mouse died at the higher tested dose (32 mg/kg). ^3^ Data from [25]. ^4^ Datum from [24]. ^5^ Datum from [28]. ^6^ Datum from [29]. IC_50_ is the concentration inhibiting 50% of protein synthesis; LD_50_ is the lethal dose for 50% of treated animals. ^§^ not available.

**Table 2:** Characteristics and efficacy of bouganin-containing ITs in pre-clinical experiments.

Bouganin	Carrier	Target	In Vitro Studies		In Vivo Studies		Ref.
			Cell Line	IC_50_ (pM) *	Animals	Survival Rate	
native	M24	CD80	Raji (Burkitt’s lymphomaL)	4.61	n.a. ^#^	n.a. ^#^	[27]
native	1G10	CD86	Raji (Burkitt’s lymphomaL)	129	n.a. ^#^	n.a. ^#^	[27]
de-bouganin	4D5MOCB	EpCAM	NIH:OVCAR-3 (Ovarian)	700	SCID mice/NIH:OVCAR-3	100%	[38]
de-bouganin	4D5MOCB	EpCAM	MCF-7 (Breast), NIH:OVCAR-3 (Ovarian)	400	HRLN nu/nu/MCF-7	70%	[42]
de-bouganin	Trastuzumab	HER2	HCC1954 (Breast)	45	CB.17 SCID mice/BT-474	83%	[48]
de-bouganin	C6.5 diabody	HER2	HCC202 (Breast)	22	n.a. ^#^	n.a. ^#^	[49]

* The IC_50_ values refer to the cell line resulted the most sensible to the IT in the referenced manuscript. ^#^ not available.
